# It’s complicated: why do tuberculosis patients not initiate or stay adherent to treatment? A qualitative study from South Africa

**DOI:** 10.1186/s12879-016-2054-5

**Published:** 2016-11-25

**Authors:** Donald Skinner, Mareli Claassens

**Affiliations:** 1 Research on Health and Society, Faculty of Medicine and Health Sciences, Stellenbosch University, Tygerberg, Cape Town South Africa; 2 Desmond Tutu TB Centre, Department of Paediatrics and Child Health, Stellenbosch University, Tygerberg, PO Box 241, Cape Town 8000 South Africa

**Keywords:** Tuberculosis, Treatment initiation, Initial loss to follow-up, South Africa, Qualitative

## Abstract

**Background:**

Individuals who test positive for active tuberculosis (TB) but do not initiate treatment present a challenge to TB programmes because they contribute to ongoing transmission within communities. To better understand why individuals do not initiate treatment, or are adherent after initiating treatment, South African respondents were approached to obtain insights as to which factors enabled and inhibited the treatment process.

**Methods:**

This qualitative work was nested in a larger study investigating initial loss to follow-up (LTFU) amongst new smear positive TB patients across five provinces of South Africa. In-depth interviews were done with 41 adherent and initial LTFU respondents.

**Results:**

Key issues contributing to initial LTFU appeared to be a poor knowledge, or low awareness of TB treatment; stigma around TB including its connection to HIV; immediate problems in the respondents’ lives particularly poverty, lack of access to transport and the need to continue working; and problems in the healthcare facilities including under resourced facilities, poor functioning health systems and negative staff attitudes. In contrast the reasons given for being adherent related to the level of illness, support received at home and healthcare facilities, a belief in the health system and positive experiences in the health service including positive attitudes from staff.

**Conclusions:**

Key changes need to be made to the healthcare system to enable patients to initiate treatment and remain adherent, but the six month regimen of daily observed treatment presents real practical and personal challenges to patients. Alternative strategies to DOTS at facility level should be investigated to bring services closer to communities to encourage patients to access care, initiate and adhere to treatment.

## Background

South Africa is one of the focus countries of the WHO with regards to TB, with an estimated incidence of 834/100 000 population in 2014, the second highest globally [1]. Even though it is a curable disease, TB is one of the main causes of mortality in South Africa [2]. Initial loss to follow-up (LTFU) after testing for TB, i.e. not initiating treatment after diagnosis, is a key concern exacerbating the TB epidemic in South Africa and internationally [3]. Studies from South Africa show an initial LTFU rate amongst newly diagnosed smear positive patients of between 17–25% [4, 5]. Qualitative enquiry as to why patients do not start treatment is scarce. From a recent systematic review investigating initial LTFU among TB patients in National TB programmes [6], out of the twenty-three selected for the review only one study, done in 2005 in Malawi [7], used in-depth qualitative interviewing techniques. This study found that hospital admissions, delay in the receipt of sputum results and the incorrect perception of healthcare workers that a patient with a negative smear could not have TB, were important reasons for initial LTFU. Subsequently few studies assessing initial LTFU included qualitative methods. A study from Cambodia [8] used in-depth interviews to ascertain enablers and inhibitors to the acceptance of treatment initiation. They found that high indirect costs, the anticipated/real side effects of TB treatment and the non-acceptance of TB diagnoses were major inhibitors to treatment initiation and therefore initial LTFU. Two studies from India [9, 10] both used semi-structured interviews for patients enrolled in the Revised National Tuberculosis Control Programme and found alcoholism, TB-related stigma, lack of TB awareness, lack of support and the fact that patients were busy with “other jobs” main contributors to initial LTFU. Therefore the reasons for initial LTFU appear varied and context specific, with extensive gaps remaining in understanding why it occurs and what the possible enablers and inhibitors are to having patients initiate treatment. A qualitative approach allows for an exploration of key issues leading to initial LTFU in relation to the context of the patients and their health services. Our aim was therefore to understand initial LTFU in newly diagnosed smear positive TB patients using in-depth qualitative interviews. In addition, we explored non-adherence to treatment. Included in this aim was the identification of enablers and inhibitors for both adherence and initial LTFU. While the focus of the research was on initial LTFU, the interviews produced considerable insights into non-adherence, leading to the aim being extended to include non-adherence.

## Methods

This study was nested within a larger study (submitted) looking at the extent of initial LTFU among TB patients. Within the larger study, qualitative research was introduced to develop an understanding of how the respondents felt about the services they received and what the personal constraints preventing them from initiating TB treatment and staying adherent were. In-depth interviews were done with patients who either had initiated treatment and had remained adherent or initially LTFU, to obtain insights from both groups as to which factors enabled and inhibited the treatment process. The interviews were done by two fieldworkers who had experience and training in qualitative interviewing. They were given additional training in the research subject matter and discussion schedule.

### Setting

TB is acknowledged by the South African government to be a major threat to the health of the South African population. In South Africa, TB services are provided at primary health care facilities by nurses and are free of charge. When sputum is collected from individuals presumed to have TB, their names are documented in a sputum register and on starting treatment they are entered into the TB register. In accordance with directly observed therapy short course (DOTS), TB patients receive medication daily at their PHC clinic or from a community treatment supporter.

### Population

The study was conducted at primary healthcare facilities (PHC) in five provinces of South Africa from October 2010 - October 2011, namely the Eastern Cape, North West, Limpopo, Mpumalanga and KwaZulu-Natal. All have endemic TB and high HIV co-infection rates. The facilities included urban, peri-urban and rural settings, but none in city centres. The communities were mostly of poor socio-economic status. Most individuals from these communities, particularly in urban or peri-urban settings, lived an accessible distance to a PHC but the dispersal of communities in rural settings increased travel requirements. Individuals were included in the study who had a smear positive result recorded in the facility sputum register of a PHC in the three months prior to the research visit, but excluding the month just prior to the visit to allow for treatment initiation. A person initially LTFU was defined as documented sputum smear positive in the sputum register, but not appearing in the TB treatment register within four weeks since diagnosis of smear positivity at the PHC.

### Sample

For this study, 41 PHC were visited. From each facility’s sputum register we selected the first ten patients diagnosed but not initiated on TB treatment and ten who had initiated treatment. Each visit to a facility lasted one day. The first patient either on treatment or initially LTFU was approached to have an in-depth interview. There was no variation in age between those included and not, but slightly more women were interviewed. Nobody refused to be interviewed.

### Interview schedule

The discussion schedule was similar for those who adhered and were initially LTFU. The key question for those who adhered was “what enabled you attending the facility to receive your test results and to begin treatment for TB”, and for those who were initially LTFU, “why you did not come back to the facility to receive your test results and potentially begin treatment for TB”. In both cases the probes covered: accessibility of the facility, experience at the facility, use of alternative health services, concerns about being diagnosed with and getting treatment for TB, difficulties generated by poverty and external events that may have affected coming for treatment. The fieldworkers were aware of the individuals listed as being on treatment and initially LTFU and focused on those issues in the interviews.

### Analysis

All the interviews were transcribed and translated into English. Both authors read ten interviews to draw out a set of themes which formed the basis of the analysis. The themes drew both on the overall aims of the study and the interview transcripts. Each theme was defined so that a common understanding of that theme could be referred to during the coding process. Once complete, the authors applied this theme list to code the interviews using Atlas ti. All interviews were read by both authors to ensure full familiarity with the data. The first author, male, comes from a social sciences and community based research background, while the second author, female, is a medical doctor with a long history in TB research. The first author is very aware of the situation of health services in South Africa, but had no contact with the facilities, patients or other contextual factors in this study. The second author who visited all the facilities and was the principal investigator of the larger study corrected for any contextual misunderstandings. Discussions took place on a regular basis to clarify misunderstandings and differences of interpretation. To validate findings interviews were reread and new insights included. The methods used were in accordance with well described qualitative literature [11, 12].

The analysis is divided into two sections, firstly dealing with inhibitors leading to initial LTFU and non-adherence and secondly enablers that facilitated initiation of treatment and adherence. The text includes quotes from the interviews to illustrate the arguments. These are linked to the community from which the respondent came and whether or not they were considered to be initially LTFU.

### Ethics

The proposal was approved by the Health Research Ethics Committee of Stellenbosch University. All participants signed an informed consent form in their own language, which was administered by the fieldworker. The letter was at a literacy level felt to be appropriate to the respondents and the interviewer checked that the respondents had understood the research and the fact that the interview would be recorded prior to them signing the letter. The recorder was switched on for the interview only after the consent letter was signed. The study was explained in the respondent’s home language by a person fluent in their language. No incentives were provided for participation.

## Results

The 41 interviews were split between those adherent to their TB treatment (22) or initially LTFU (19). The sample was distributed across the provinces, eight respondents from the Eastern Cape (six who were adherent: two initially LTFU), five from KwaZulu-Natal (3:2), nine from Limpopo (4:5), fifteen from Mpumalanga (6:9) and four from Northwest province (3:1). The results are presented in two sections below, first describing the inhibitors to treatment initiation and adherence and then the factors enabling adherence.

### Inhibitors leading to initial loss to follow-up (LTFU) and non-adherence

Respondents’ reasons for not starting treatment centred on the challenges of accessing treatment, poverty, stigma and the difficulty of staying on treatment for an extended period.

### Not feeling ill

Respondents, who did not feel ill and were tested but did not have any symptoms, stated that they did not believe the test results, especially when seeing TB patients who were very ill. Many of these respondents had not presented themselves at the facility to be tested. Facility staff visited the households of patients already on treatment to screen others residing there for undiagnosed TB.

*Yes they came with my result but I was not sure if those were mine. …….. I thought they might have mixed them. …………. Because I see myself that I am still strong and big (Limpopo initial LTFU)*

Even for those who initiated treatment, as they felt better and their symptoms reduced, the motivation to stay on treatment reduced. Respondents reflected that in previous incidents of TB usually two months after starting treatment, the motivation to stay on the medication dropped, and treatment fatigue increased. Some reported stopping treatment when the symptoms disappeared, but starting again when the symptoms returned.

*For about three months or four then I started feeling those symptoms and that is when I started to take it again (Limpopo initial LTFU)*

### Accessing facilities, problems of distance and transport

Where there was no community DOTS service, patients had to make the journey daily.

*… if you are not fit enough it can even take 3 hours. …….. Too much, too much everything is a problem there are a lot of defaulters at [] because of the distance they are complaining because it is too far that is why there are so many defaulters. (North West initial LTFU)*

The distribution of health facilities in urban areas were mostly situated centrally so homes would be within a 20 min walk. Problems did occur where communities were dispersed, such as in rural areas, as many patients had to travel longer distances. When the weather was bad, particularly when raining, accessing services became harder. In discussions with PHC staff it was reported that mobile services have been established to facilitate access, but as the communities got more scattered even this service was difficult to maintain. Patients far away either had to walk long distances or had to pay a taxi or bus fare. Given that few TB patients are able to work these costs made adherence difficult, especially with competing costs such as food. When patients were really ill, the symptoms made walking difficult with one person commenting that the walk would take them 45 min or even three hours leaving them feeling dizzy.

### Conflict with work

Sometimes respondents were referred for treatment, but because of work related issues, particularly work hours and the place of work, they could not access care. Respondents were concerned that if they waited for care they would be late for work and they would lose their employment. They were also afraid to tell their employers about their diagnosis as this could cost them their position. The scenario created a vicious circle because the person who was not treated would get sicker and eventually lose their job anyway. Some who stayed on treatment lost wages for that period. One solution was to go to the facility before or after work, when there may be less people in the queue. This depended on working hours, distance to work, sensitivity of employers, hours when the facility operated and the motivation of the patients.

*No I did not start ………. I did not go back to them because of my work schedule because I work from 07:00 till 16:30. There they open from 08:00 until 16:00 and on week end they are closed… (Mpumalanga initial LTFU)*

### Problems experienced at the facility

A lack of organisation at the facilities causing delays in service and queues was reported in varying degrees across the five provinces. This principally impacted on the daily DOTS programme, but is likely to also have affected all other visits. Where the facilities had separate queues or rooms for patients with TB, queues moved fast and patients received their treatment without delays. However, there were facilities where patients had to wait considerable periods to get treatment and queues were reported to be between 4 h to taking all day. Patients felt obliged to get to the facility very early and wait for the facility to open. There was a sense of despondency regarding the slow service. Problems in the facilities often arose from understaffing especially in relation to the size of the population being served.

*There is a queue but you must be patient to everything because there are other people that go there. You just need to wake up early at about 06:30 and by the time they open up at about 07:20 you should be there. (Eastern Cape Adherent)*

Negative staff attitudes created problems in some facilities, with one respondent feeling that the staff did not care and patients just had to wait. So while the staff of some facilities would act to make sure that those coming for treatment got seen quickly and efficiently, in other facilities problems were recorded. A further and more serious inhibitor was the reporting of abusive attitudes by staff. Complaints included that staff sent a patient away when they assumed that he was not taking treatment, treat patients like children and in a derogatory manner, blame patients for problems for which they are not responsible and for shouting and swearing at patients. These raised anger and irritation at the services. While the fear may motivate patients to remain on treatment, it can make it difficult for them to return if they did not initiate treatment. Over time it may undermine respect for the professional status of the staff and the power of medical authority. It is understood that many patients generate high levels of frustration in the staff, and problems in the levels of staffing and the lack of support for the staff also raise staff frustrations.

*I would like to continue with my treatment I just don’t want to be shouted at because that is also working me on my nerves I wish they can talk to this nurse because she is making me angry sometimes. (Eastern Cape Adherent)*

This was not the universal experience and many respondents spoke of having positive experiences at the facilities.

There were distinct problems around the testing of patients and particularly around informing them of their results. Amongst the issues raised was that they were not informed that they had TB at the facility and therefore did not start TB treatment. Some were tested without knowing what the test was for, so were not really aware of needing to return for treatment. Even if the results were positive many only found out one or two months later. In some cases patients would return for their results on the appointed day and the results would not be available. At one facility the respondents reported that the staff made TB treatment cards for patients to ensure they knew when to return for treatment and those who tested positive were followed-up if they were late in returning.

At other facilities there were misunderstandings about treatment, for example patients were not started on the continuation phase treatment on time as many of the nurses were not aware of the need for this.

*I went to the clinic for the test and I did it and I brought the jars they gave me, I waited up to a couple of days as they told me, when I came back they told me to spit again because they lost my results of TB. I did it again but the thing is, it took so long to came back and I was going to lose my patience because for me that was so serious, I wanted to know exactly what is really the matter with me. I looked at myself losing weight, tiredness, pains in my body. (Mpumalanga Adherent)*

### Stigma

Stigma did not appear to be a major problem amongst all the respondents but it did exist. The efforts of some facilities to create a discrete service for TB patients, or enabling them rapidly collecting their medication and leaving, reduced stigma or at least limited the potential exposure to stigma. It also decreased the possibility of transmitting TB to other patients. However interventions are still required in the communities to reduce stigma as there were experiences of stigma reported. One respondent said his girlfriend threw him out and his family refused to have him in their house, so he was reduced to steal food to survive.

*You see the thing is for me to get food at this house my niece must steal it for me because her mother doesn’t want me. Even at the clinic they know that. (Eastern Cape Adherent)*

Respondents felt less stigmatised if someone close to them was on TB treatment. This provided a space to share fears and concerns. It meant that someone else understood and supported them.

*No I don’t because people don’t have a problem of TB, but they do when it comes to HIV they are scared of HIV. But even when I go to the clinic to collect my ARV we do talk and also my husband is taking them (Kwazulu Natal Adherent)*

Some respondents spoke defiantly about the stigma, expressing real anger at those who labelled them and discriminated against them. There was resistance with respondents stating with resolve that they would continue despite the stigma. It appeared to be an ongoing battle in the minds of people and it took emotional strength to resist the stigmatising comments. The resolution of this conflict will depend on the levels of stigma in the community, the other support systems of the patient, their dependence on the community for support and the patient’s own ego strengths. Those with more severe symptoms said that when they were feeling very ill, stigma was not an inhibitor. They just wanted to take their medication to get over the illness.

*I do have that problem sometimes but I told myself that this is my life. I don’t mind what other people are saying because TB is everywhere. People will lose weight and gain it again it’s not going to help if I am going stay inside the house. (Eastern Cape Adherent)*

### Association between TB and human immunodeficiency virus (HIV) infection

The connection between TB and HIV presents an additional challenge, as the fear and stigma attached to HIV was seen as being transferred to TB. It does appear that HIV carries a higher stigma than TB as HIV is not curable. Community members are seen as looking for signs that a person is HIV positive and increasingly TB was been seen as indicating a dual diagnosis of HIV, stemming from the high levels of co-infection.

*Most of them are very scared to test for TB because you know that TB is related to HIV, immediately when you tell them that they have TB they say “oh my god, which means I already have HIV”. (North West Adherent)*

### Side effects

Respondents reported side effects such as hunger and vomiting if they took the medication without food. It was a particular inhibitor in poor communities and amongst the unemployed as food was often difficult to obtain, especially when the TB patient had to give up work to be able to collect their medication.

*Also if you don’t have anything to eat and eating plays a major role in this it boosts this medication, so if you don’t have food this medication causes a problem in your body. (Limpopo initial LTFU)*

No other major side effects were reported as contributing to initial LTFU. In those cases where side effects occurred, some patients’ treatments were adapted in order to reduce side effects. The capacity to provide this assistance depended on the service providers at the facilities and was not easy since facilities have a standard treatment regimen. There was a secondary cautionary theme of distrust that the medication would not be effective. Some commented on such cases where the treatment did not work or where people repeatedly kept getting TB.

### Some want to drink and smoke

The injunction not to drink and the implications of drinking while on treatment made the treatment unpopular with especially young men. Respondents reported that alcohol tends to form a centre for social interaction in many social circles; so not being able to drink segregates individuals from their peer grouping. Even if they did start taking medication, when they were feeling ill early in the treatment process, once they are better there is a desire to re-join their social circles.

*Oh, sometimes when he was drinking we used to tell him that he should stop drinking and to stop smoking because they don't go together with the medication. He eventually stopped smoking I don't know about the drinking (Laughing). (North West Adherent)*

### Personal problems attributed to initial LTFU

Respondents spoke about others who did not initiate treatment or who would not complete treatment, saying that they were lazy or stupid, and as not valuing their lives. Nobody stated that they had given up on life and therefore stopped treatment, but it was assigned to others as a reason for not completing treatment. It is difficult to assess how valid this is as a reason and it will require further research. However, the levels of deprivation and hopelessness that many people reported could contribute to such an attitude.

*I think they are lazy to take them because of every day treatment some must take it in the morning but they will put it in the toilet and flush them or under the bed. (Mpumalanga initial LTFU)*

### Enablers that facilitated initiation of treatment and adherence

The motivations that respondents who had adhered to treatment were largely consistent across all the regions and reflected a need to follow the rules and a desire to get well, especially with the twin threats of multidrug resistant TB (MDR-TB) and HIV infection.

### Following instructions

A strong desire to follow the instructions of the medical staff was a key theme. The power of the norms of the medical model and of the status of the health professionals carried considerable weight. Respondents stated that since they had received the instructions they felt obliged to follow them.

*I was told that I have it [TB] and I understood that I went there because I don’t know what is going on with me. So if they told me that this is the problem I just have to accept it and take my treatment that is all. (Limpopo initial LTFU)*

### Feeling very ill

The level of illness and disability resulting from the symptoms provided a strong motivation to continue with treatment. TB left many of the patients in considerable pain and unable to complete basic personal tasks. They felt they had no options but to adhere. Avoiding hospitalisation was a further motivation.

*It was the pain I was feeling I was in pain and so that is why I thought I should go for treatment I realized nothing else will help me other than the treatment, I have to take my treatment until I complete it. The other thing is I am so scared of really sick people I don't want a person to be so sick that they get pushed around in wheelbarrows and things like that, so that is why it is important for me to get help while I can still walk on my own two feet to get help from the clinic. (North West Adherent)*

The need to be cured is the second half of the motivation. Respondents had seen the treatment work and wanted to be healthy themselves. The direct threat of TB meant that respondents feared death and wanted to take treatment to stay alive to be able to care for those they loved. Some respondents reported having stopped treatment before, but now wanted to complete their treatment.

*because I want to see myself better again like before that is why I take it because I know if I don’t take them I will die and live my children alone. (Mpumalanga Adherent)*

A strong message from those who were adherent was the positive impact of the treatment and the return to health of other people around. Despite some inhibitors as expressed above, the dominant understanding was that treatment would lead to cure. They spoke of being able to complete chores and that their clothes now fitted them.

### Avoiding the development of MDR- or extremely drug resistant (XDR)-TB

The increasing presence of MDR- and XDR-TB has increased the motivation amongst many to seek treatment and complete treatment, with the belief that untreated TB becomes MDR-TB. Besides the more painful and complicated treatment, a greater stigma and fear was associated with MDR-TB.

*there different kinds of TB out there like MDR and XDR and once you have this TB and not take your treatment you will have a problem whereby they will tell you that the treatment is not working properly in your (Eastern Cape Adherent)*

There was also a mistaken belief reported by one respondent that untreated TB can lead to HIV, which motivated those who reported this to adhere to treatment. This is more likely to arise from the continued linking of the two diseases.

### Experiences and knowledge of other illnesses

Respondents used recovery from other illnesses as a reason for taking treatment. Most were able to generalise from the successes of treatment of infectious diseases and other health problems to the likely success of TB treatment. There was also an underlying message that TB is treatable, unlike HIV, so they needed to take advantage of the treatment and get well. This distinction was strongly felt as most of these communities have been strongly affected by HIV.

### Being HIV positive

Respondents who were HIV positive spoke of an additional motivation to be on treatment as they felt that their life was under a more direct threat. The additional inputs associated with being on antiretroviral treatment meant that they understood the importance of adherence better. They also had additional contacts with health professionals especially in the early phases of treatment.

*And if they are saying its 8 month I must take it so that I can be better again because at the end of the day TB is curable. Yes there are those who are not curable but if you follow your treatment everything will be fine. Even if its treatment for AIDS because each treatment has its own way of working in your body. (Limpopo Adherent)*

### Support

Support from family, friends and community played a role in people being adherent. This was key on the emotional level. If supported the person felt encouraged and to have a reason to continue. At the practical level: others assisted the patient getting to the facility to get medication and in providing food so that they could take the pills. Families were reported to encourage patients to return to treatment. Conversely the absence of support was also seen as a reason for not initiating or staying on treatment. Support also led to respondents taking treatment to avoid infecting those they lived with and cared about.

*I told myself that if I had TB I will accept it and just take my treatment. Because there are many of us here at this house if I stay and not take my treatment I will infect others. (Eastern Cape Adherent)*

### Positive relationships with the facility staff

As noted above, most respondents were positive about the staff and their treatment at the health facilities. They felt they could trust healthcare workers with their management and to be confidential about their diagnosis. Where the staff were positive and supportive, and thereby able to develop a good relationship with the patients, this served to encourage people to attend as the patient felt supported and encouraged.

## Discussion

This paper discusses inhibitors preventing patients from presenting at primary healthcare facilities in South Africa to initiate treatment and which lead to poor adherence.

Patient knowledge and beliefs are shown to be important in both presenting for treatment once diagnosed and in adherence once on treatment, especially where the demands of treatment conflict with lifestyle, for instance the wish to stop treatment when feeling better, the belief that treatment is not needed before becoming seriously ill or the wish to stop treatment in order to return to aspects of a previous lifestyle, particularly drinking alcohol. This is similar to results from other sites such as Bali [13] where it was shown that respondents did not believe the early symptoms of TB to be serious and Timor Leste, where respondents stopped treatment because they were feeling better [14]. For patients who are HIV positive, adherence on antiretroviral treatment (ART) could be improved by addressing factors such as self-efficacy and patient beliefs regarding the efficacy and safety of ART [15]. It therefore seems that for both TB treatment and ART adherence, psychological factors on the individual level is of major importance especially in a setting like South Africa where both diseases are of epidemic proportion and many TB patients are co-infected with HIV. In such co-infected patients, pill burden, economic constraints, side effects, lack of food, stigma and poor communication with healthcare staff can be additional barriers to treatment and should be addressed as well [16].

Although not identified in this study as a serious problem, the related area of stigma and fear around TB needs to be monitored, as was shown previously in the Western Cape [17] since it is getting more important as TB increasingly becomes linked to HIV in the minds of community members [18, 19]. A number of patients spoke with great distress about being rejected by their family and community as a result of being diagnosed with TB. This contrasts with patients’ need for support if they are to initiate and remain on treatment. Possible enablers to engage such patients to initiate treatment could be peer-based support groups, family counseling and community treatment supporters.

Poverty leading to a lack of food or not being able to access the healthcare facility remained a challenge to both initial LTFU and non-adherence in our study and was demonstrated in previous studies from South Africa [20] and Pakistan [21]. Severe symptoms make the situation worse as the really ill patients are unable to do much for themselves: they have trouble walking even short distances to facilities and become reliant on others so there is an additional need to access transport. This is similar to what was reported from Timor Leste [14]. The impact of having to collect medication daily from the healthcare facility on a person’s capacity to work is having a deterrent effect with many respondents stating that they cannot afford to come late for work, or reveal their status to their employers, for fear of losing the employment. The same inhibitor was reported from China [22], Cambodia [8] and Pakistan [21] although work place DOTS could be effectively delivered with high adherence rates [23] and would be a feasible solution with employer buy-in.

The healthcare facilities appeared to offer good services and provide support for many patients. However, there did remain many inhibitors to initiating treatment, such as the restricted opening times noted above. This duality was also reported from other countries, albeit not in patients initially LTFU [14, 24–26]. Some spoke highly of the support that they received and the efforts of the staff to give their treatment quickly and efficiently. This contrasted with other facilities where patients stated that they had to wait up to four or even seven hours to be assisted, where nurses were rude and condescending to them and where they were fearful to go. Similar conditions were seen in Pakistan [21].

All of these issues can be remedied, for example the retraining of staff to improve their patient interaction may facilitate a more welcoming environment and having a separate queue or fast track for TB patients could be introduced at all facilities. A concern does remain over the provision of adequate resources to facilities, especially staffing. This feeds into the negative staff attitudes and makes the implementation of new ideas difficult. Alternative approaches to get treatment to patients may have to be considered. Key to some of the problems is the directly observed treatment, short-course (DOTS) programme which requires patients to come to facilities daily at most facilities. This directly impacts on patients who have mobility problems or have to work [8, 21] and put facilities under additional pressure. It is difficult to provide communities with resources that will allow them to initiate treatment or at least get to facilities. While there are advantages to DOTS, these negative implications have to be taken into account especially since DOTS have been shown not to resolve poor adherence [27]. A larger community based or mobile service should be a high priority, for instance in Cambodia where patients who were severely ill or faced challenges regarding transport noted that home-based care was an acceptable alternative [8], similar to what was found in Swaziland [28] and in Uganda [29], where TB patients indicated that community-based care was preferred to hospital or clinic-based care. In Tanzania, it was also shown that a patient-centred treatment approach had better treatment success rates compared to daily health facility DOTS [30] and that treatment supporters who live close to the patient facilitate adherence [31].

### Limitations

It was not possible to implement purposive sampling as the fieldworkers were reliant on the facility staff and the sampling method of the larger study to access potential interviewees. So while the sample is widely dispersed across the country, the numbers remain small and it is difficult to ascertain how representative they are of the general population of TB patients. The sample size was guided by the sampling procedure used, rather than the process of reaching data saturation. However, there was a consistency across the interviews, so we feel it is unlikely that we missed major themes. We were not able to triangulate findings against other data as the questions were different in many respects from other components of the study in which this qualitative research is nested. As the sample was spread across five provinces of South Africa we were unable to return to check findings. By piggybacking these interviews on top of an existing national study we were provided with an unique opportunity to draw a sample from a large population frame.

## Conclusions

Initial loss to follow-up amongst newly diagnosed smear positive TB patients is a key concern for the programmatic management of TB in high burden countries such as South Africa. Healthcare staff and programme managers should address inhibitors hindering patients to initiate and adhere to treatment, such as patient knowledge and beliefs, stigma and fear, and the inability to access care due to poverty. Healthcare staff must be made aware that their attitudes and practices influence patients’ willingness to initiate and adhere to treatment. Alternative strategies to DOTS at facilities must be investigated to encourage patients to access care, initiate and adhere to treatment.

## Abbreviations

AIDS: Acquired immunodeficiency syndrome
ARV: Antiretroviral
DOTS: Directly observed treatment, short-course
HIV: Human immunodeficiency virus
LTFU: Loss to follow-up
MDR-TB: Multidrug resistant tuberculosis
PHC: Primary healthcare facilities
TB: Tuberculosis
WHO: World Health Organization
XDR-TB: Extremely drug resistant tuberculosis
